# Detection of thrombus size and protein content by *ex vivo* magnetization transfer and diffusion weighted MRI

**DOI:** 10.1186/1532-429X-14-45

**Published:** 2012-06-25

**Authors:** Alkystis Phinikaridou, Ye Qiao, Nick Giordano, James A Hamilton

**Affiliations:** 1 Division of Imaging Sciences and Biomedical Engineering, King’s College London, London, UK; 2 Department of Physiology and Biophysics, Boston University School of Medicine, Boston, MA, USA; 3 The Russell H. Morgan Department of Radiology and Radiological Sciences, The Johns Hopkins Hospital, Baltimore, MD, USA; 4 Department of Biomedical Engineering, Boston University, Boston, MA, USA

## Abstract

**Background:**

To utilize a rabbit model of plaque disruption to assess the accuracy of different magnetic resonance sequences [T1-weighted (T1W), T2-weighted (T2W), magnetization transfer (MT) and diffusion weighting (DW)] at 11.7 T for the *ex vivo* detection of size and composition of thrombus associated with disrupted plaques.

**Methods:**

Atherosclerosis was induced in the aorta of male New Zealand White rabbits (n = 17) by endothelial denudation and high-cholesterol diet. Subsequently, plaque disruption was induced by pharmacological triggering. Segments of infra-renal aorta were excised fixed in formalin and examined by *ex vivo* magnetic resonance imaging (MRI) at 11.7 T and histology.

**Results:**

MRI at 11.7 T showed that: *(i)* magnetization transfer contrast (MTC) and diffusion weighted images (DWI) detected thrombus with higher sensitivity compared to T1W and T2W images [sensitivity: MTC = 88.2%, DWI = 76.5%, T1W = 66.6% and T2W = 43.7%, *P* < 0.001]. Similarly, the contrast-to-noise (CNR) between the thrombus and the underlying plaque was superior on the MTC and DWI images [CNR: MTC = 8.5 ± 1.1, DWI = 6.0 ± 0.8, T1W = 1.8 ± 0.5, T2W = 3.0 ± 1.0, *P* < 0.001]; *(ii)* MTC and DWI provided a more accurate detection of thrombus area with histology as the gold-standard [underestimation of 6% (MTC) and 17.6% (DWI) compared to an overestimation of thrombus area of 53.7% and 46.4% on T1W and T2W images, respectively]; *(iii)* the percent magnetization transfer rate (MTR) correlated with the fibrin (*r* = 0.73, *P* = 0.003) and collagen (*r* = 0.9, *P* = 0.004) content of the thrombus.

**Conclusions:**

The conspicuity of the thrombus was increased on MTC and DW compared to T1W and T2W images. Changes in the %MTR and apparent diffusion coefficient can be used to identify the organization stage of the thrombus.

## Background

Acute thrombus formation after coronary plaque disruption has been recognized as the main cause of cardiovascular events [1-4]. MRI studies of thrombosis in experimental animals both *in vitro*[5] and *in vivo*[6], and histological evaluations of arteries from sudden death victims [7,8], have revealed compositional changes that occurred over variable time periods (days to weeks) during thrombus evolution. Coronary thrombi aspirated from patients with acute ST-segment elevation myocardial infarction have been estimated by histology to be days or weeks old in 50% of the cases [9]. Thrombus age appears to be an independent predictor of long-term mortality [10]. For a large number of patients presenting with symptoms suggestive of acute coronary symptom (ACS), diagnosis of the underlying cause is often unclear [11]. Thus, direct and non-invasive thrombus imaging may be beneficial for medical decision-making, as it would provide information whether blood flow occlusion is due to a highly stenotic plaque or whether plaque rupture and thrombosis has already occurred.

MRI has been successfully applied for detection of thrombus in hematoma [12,13], venous thrombosis [14,15], intraplaque hemorrhage [16,17], and arteries [6,18,19] by exploiting the T1-shortening effects of meth-hemoglobin. However, meth-hemoglobin is usually formed in the acute stages of thrombosis because of the local hypoxic environment of occlusive thrombus but is not present during the chronic stage. To further improve the ability of MRI to image thrombus, fibrin [20-26], platelet [27-29] and α2-antiplasmin–based [30] targeted contrast agents have been developed, but none of these agents has been approved for clinical use. Therefore, a non-invasive, non-contrast enhanced imaging method to image thrombus characteristics may have significant diagnostic and therapeutic value.

In our previous studies, mural thrombus associated with plaque disruption in the rabbit aorta was detected *in vivo* with T1W and T2W images [25,31] and thrombus visualization was later improved with the use of a fibrin binding contrast agent [20]. DWI has been shown to detect thrombus [18,32-35], and MT could be a promising, yet unexplored, approach to detect regions of organized proteins [36,37]. Because of the protein content of thrombus and the differences in the diffusivity of water molecules depending on the local microenvironments, we hypothesized that MT and DWI could be used to generate endogenous contrast for imaging thrombus without the need of targeted agents. The aims of this study were to: *(i)* compare the imaging characteristics of thrombus on multi-contrast MR images (MT, DWI, T1W, T2W) acquired *ex vivo* at 11.7 T, *(ii)* test the sensitivity of MT and DWI for non-contrast enhanced detection of thrombus *ex vivo,* and *(iii)* assess the accuracy of MT and DWI for detection of thrombus size and composition compared to the standard T1W and T2W image sequences and using histology as the gold-standard.

## Methods

### Animal model

Atherosclerosis was induced in adult male (n = 17) New Zealand White rabbits (Charles River Laboratories, Wilmington, MA) as previously described [31,38]. Briefly, rabbits were fed a 1% cholesterol diet (PharmaServe, Framingham, MA) for 2 weeks before and 6 weeks after balloon injury of the abdominal aorta, followed by 4 weeks of normal diet. Plaque disruption and thrombosis was induced with Russell’s viper venom (0.15 mg/kg, IP, Enzyme Research, South Bend, IN) followed by histamine 30 min later (0.02 mg/kg, IV, Sigma-Aldrich, St. Louis, MO). This procedure was performed twice within 48 h. Finally, 24 h after the last pharmacological triggering, the animals were sacrificed with sodium pentobarbital (100 mg/kg, IV). Heparin was infused to inhibit postmortem coagulation. The aortas, from the renal branches to the iliac bifurcation, were harvested, sectioned and catalogued. The segments were fixed in 10% formalin overnight and then transferred in PBS for *ex vivo* MRI followed by histology. All experiments were performed under the approval of the Institutional Animal Care and Use Committee on Animal Investigations.

### *Ex vivo* MRI of rabbit thrombus at 11.7 T

The specimens used for this *ex vivo* study were derived from our previous work that focused on *in vivo* imaging of atherosclerosis [31]. *Ex vivo* MRI was performed in a vertical-bore Bruker Avance spectrometer (11.7-Tesla) fitted with gradient coils (bore size = 89 mm; maximum gradient strength = 906.6 mT/m). Aortic segments were imaged in phosphate-buffer saline (PBS) using a 10 mm birdcage transmitter/receiver coil. During data acquisition the samples were maintained at 37°C, using a thermocouple-heating element. 2D T2W spin-echo images were acquired with: TR = 3 s, TE = 30 ms, averages = 128, slice thickness = 1.0 mm, matrix = 128 × 128, FOV = 6.5 mm (resolution = 50x50μm), scan time = 7 min. 2D T1W spoiled-gradient-echo images with and without MT were acquired with: TR = 330 ms, TE = 4 ms, flip angle = 30°, averages = 192, slice thickness = 0.5 mm, matrix = 256 × 256, FOV = 6.5 mm (resolution = 25 × 25μm), scan time = 1 h. A Gaussian saturation MT pre-pulse was applied 10000 Hz off-resonance with duration of 12 ms and power of 20μT once every TR. These conditions were previously optimized in our lab using phantoms and carotid endarterectomy specimens [37]. MT employs an off-resonance pulse that selectively saturates the water molecules bound to macromolecules compared to those in the free water pool, resulting in images with reduced signal intensity that is proportional to the amount of macromolecules [39-41]. Finally, 2D DW images were acquired with: TR = 1 s, TE = 25 ms, Δ = 12.6 ms, δ = 5 ms, averages = 32, slice thickness = 1 mm, matrix = 128 × 128, and FOV = 6.5 mm (spatial resolution = 50 × 50 μm), scan time = 6.5 h. The apparent diffusion coefficient (ADC) was calculated from seven b-values = 0, 196, 442, 637, 867, 1770 and 2409 s/mm^2^. For DW imaging a b-value = 442 s/mm^2^ created the best contrast between the thrombus and the vessel wall and was used to calculate the thrombus area. T2 relaxation was determined using a Carr-Purcell-Meiboom-Gill sequence with TR = 3 s and ten different echo times with echo spacing of 6.3 ms, scan time = 3.3 h. T1 relaxation was determined using a spin–echo sequence with 10 different TR values ranging from 100–8000 ms, scan time = 7 h. The rest of the acquisition parameters were similar to those listed for the T2W and T1W images above.

### Histology

Aortic segments were embedded in tissue freezing medium and stored at -80°C. Serial 10 μm thick cross-sections were used for Masson’s trichrome staining to detect collagen and immunohistochemistry to detect fibrin. Immunohistochemistry was performed by the avidin-biotin-peroxidase method (Vector Laboratories, Burlingame, CA). An anti-rabbit mouse monoclonal antibody for fibrin (American Diagnostica Inc, Stamford, CT, No. 350) was used. Disrupted rabbit aortic plaques contained an overlying thrombus similarly to what has been observed for human coronary plaques [42].

### Data analysis

MR images acquired at 11.7 T were processed with the Paravision 3.02 software (Bruker, Ettlingen, Germany). Images acquired at 3 T were processed with Osirix (OsiriX Foundation, Geneva, Switzerland). Regions of interest (ROI) encompassing the thrombi and plaques were manually segmented on all sequences, and the mean signal intensity (SI) of each ROI was recorded. The signal-to-noise ratio (SNR) of thrombus and plaque was calculated as SNR = (SI_component_-SI_noise_)/SD_noise_ Noise was determined within an ROI drawn outside of the specimen in the buffer. CNR between thrombus and plaque was calculated as (SI_thrombus_-SI_plaque_)/SD_noise_. All values were expressed as absolute numbers. Furthermore, images acquired with and without magnetization transfer were used to calculate the percent magnetization transfer rate (% MTR) based on the formula: % MTR = [(SI _without MT_ - SI _with MT_)/SI _without MT_]* 100. The same ROIs were used to calculate the apparent diffusion coefficient (ADC) based on the equation ADC = ln(S_0_-S_1_)/b_1_-b_0_ (mm^2^/s), and the T1 and T2 relaxation times using the DW, T1 and T2 images, respectively.

Thrombus cross-sectional area (mm^2^) in rabbit specimens was measured on trichrome stained sections and on *ex vivo* MR images by computerized planimetry (ImageJ, NIH). In the case of MT, the thrombus area was calculated using the magnetization contrast images (MTC) generated by subtracting the image with MT from that without MT. Computer-assisted color image analysis was used to quantify the collagen area on trichrome stained sections and the positive areas for fibrin on immunohistological sections, which were expressed as percentages of the total thrombus area.

For registration of the *ex vivo* MR images of rabbit specimens and histological sections, the distance of the proximal end of each segment, the gross morphology, and internal plaque/thrombus landmarks visible on both the MR images and histology were used as references.

### Statistical analysis

The Statistical Package for the Social Sciences 18.0 (SPSS Inc.) was used and data are presented as mean ± SD. Probability values of *P* < 0.05 were considered significant. For 2-group comparisons, continuous variables were compared with a Student’s *t*-test. The correlations between continuous variables were assessed using Pearson’s correlation. Multiple group comparisons were performed by one-way analysis of variance (Anova). The receiver operating characteristic curve was used to compare the accuracy of different MRI sequences for detecting the presence or absence of thrombi. Bland-Altman plots were used to assess the agreement between different MRI sequences and histology in measuring thrombus area. Two independent observers (A.P and N.G) analyzed the MR images and were blinded to all histological data. The inter-observer variability was assessed using the intra-class correlation coefficient (ICC).

## Results

### Histological classification of rabbit plaques

Histology revealed that all animals had aortic atherosclerotic plaques and that 9 out of 17 rabbits (53%) had luminal thrombi. Of a total of 59 plaques studied, 34 had superimposed thrombosis whereas 25 did not have any evidence of thrombus.

### Imaging characteristics of thrombus on multi-contrast MR images acquired at 11.7 T

**Figure 1:**
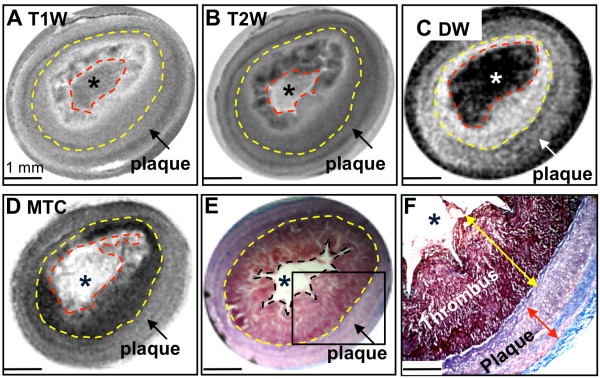
**Diffusion weighted and magnetization transfer contrast images distinguish the thrombus from the underlying plaque. A**, T1W image. **B**, T2W image. **C**, DW image (b value 450 s/mm^2^). **D**, MTC image. **E-F**, Low and high power Masson’s trichrome staining, respectively. The area of the thrombus (encompassed within the doted lines) and the contours between the thrombus and the plaque (yellow line) are more clearly seen on the DW (high signal) and on the MTC (low signal) images. The presence of a platelet and fibrin-rich thrombus superimposed on the plaque is verified histologically. Asterisks indicate the lumen.

Multi-contrast images of a disrupted plaque with a superimposed thrombus are illustrated in Figure 1. The T1W (Figure 1A) and T2W (Figure 1B) images revealed thickening of the vessel wall but did not discriminate the thrombus from the underlying plaque. By comparison, DW images (Figure 1C; ADC_thrombus_ = 0.58 × 10^-3^ mm^2^/s versus ADC_plaque_ = 0.25 × 10^-3^ mm^2^/s) and MTC images (Figure 1D; MTR_thrombus_ = 42.6% versus MTR_plaque_ = 27.4%) increased the conspicuity between the thrombus and the underlying plaque/vessel wall. MRI assignments were corroborated by histopathology (Figure 1E and 1F), which illustrated a platelet- and fibrin-rich thrombus overlying the plaque.

MTC and DWI at 11.7 T provide superior contrast between the thrombus and the underlying plaque and a more accurate assessment of thrombus size compared to T1W and T2W images.

**Figure 2:**
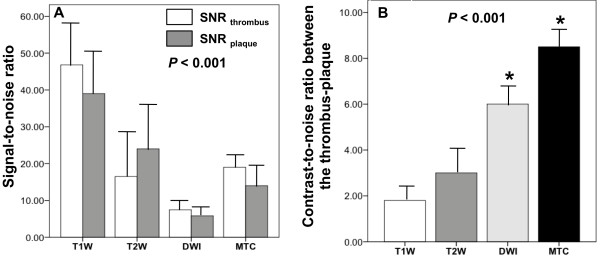
**Comparison of the SNR and CNR of different MRI sequences in detecting white-thrombi. A**, Bar graph showing that all MRI sequences provide sufficient SNR for both the thrombus and the plaque. **B**, Bar graph showing that the thrombus was better discriminated from the underlying plaque on MTC and DW images that showed higher CNR compared to T1W and T2Wimages. SNR: signal-to-noise ratio, CNR: contrast-to-noise ratio.

Quantitative assessment of the SNR for the thrombus and the plaque showed that all image sequences provided sufficient SNR for both components (Figure 2A). However, the CNR between the thrombus and the underlying plaque was significantly higher on MTC (CNR = 8.5 ± 1.1) and DW (CNR = 6.0 ± 0.8) images compared to T1W (CNR = 1.8 ± 0.5) and T2W (CNR = 3.0 ± 1.0) images (Figure 2B).

Of the 34 thrombi identified on histology, *ex vivo* MTC images correctly identified 30 (sensitivity = 88.2%), DW images 25 (sensitivity = 76.5%), T1W 21 (sensitivity = 66.6%), and T2W images 15 (sensitivity = 43.7%). All MRI sequences accurately reported the absence of thrombi (specificity = 100%). Inter-observer variability revealed good reproducibility for the measurement of thrombus area using MTC (ICC = 0.92) and DW images (ICC = 0.90). However, there was a low inter-observer agreement when using the T1W images (ICC = 0.55) and only a moderate agreement when using the T2W (ICC = 0.60) images.

**Figure 3:**
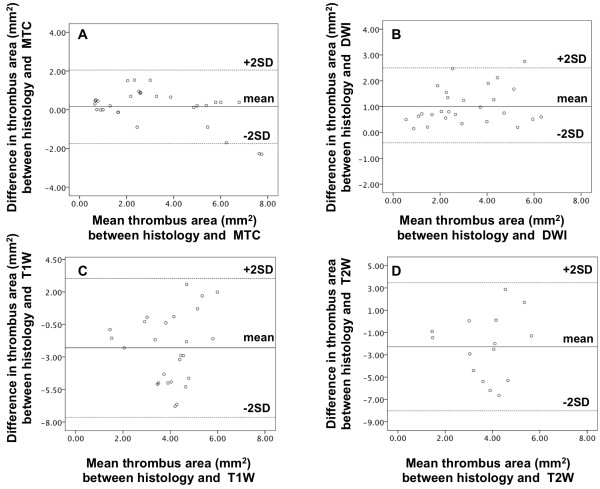
**Bland-Altman plots compare the assessment of thrombus area on different MRI sequences and histology. A**, MTC and **B**, DW images tend to underestimate the thrombus area. **C**, T1W and **D**, T2W images tend to overestimate the thrombus area. Although the area of the thrombus measured on different MRI sequences was within the 95% limit of agreement, the level of agreement was higher for MTC (A) and DW (B) images as indicated by the tighter clustering of the data points around the median value. Conversely, larger differences were observed in the area of the thrombus calculated using the T1W (C) and T2W images (D) as compared to histology (scattered data points). Furthermore, there was a trend for MTC and DWI to slightly underestimate the area of the thrombus compared to histology (6% and 17.6%, respectively). Conversely, the thrombus area was overestimated on T1W images (53.7%) and T2W images (46.4%).

The Bland-Altman analysis was applied to test the agreement in thrombus area calculated using different MRI sequences and histology (Figure 3). Although the area of the thrombus measured on different MRI sequences (Figure 3A-3D) was within the 95% limit of agreement, the level of agreement was higher for MTC (Figure 3A) and DW (Figure 3B) images, as indicated by the tighter clustering of the data points around the median value. Conversely, larger differences were observed in the area of the thrombus calculated using the T1W (Figure 3C) and T2W images (Figure 3D) as compared to histology (scattered data points). Furthermore, there was a trend for MTC and DWI to slightly underestimate the area of the thrombus compared to histology (6% and 17.6%, respectively). Conversely, the thrombus area was overestimated on T1W images (53.7%) and T2W images (46.4%).

**Figure 4:**
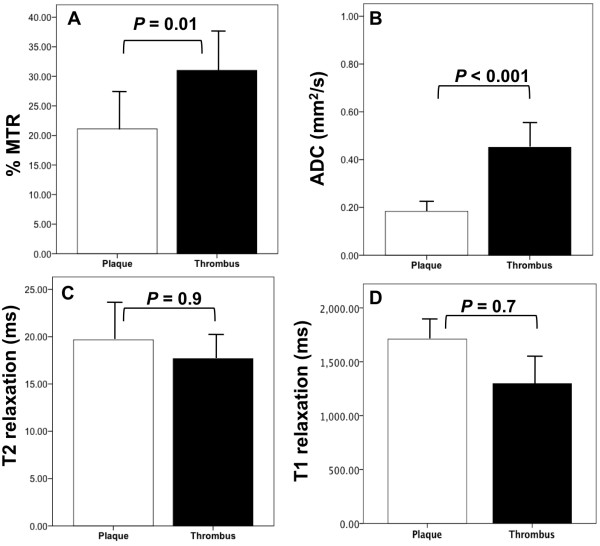
**Comparison of the magnetization transfer, apparent diffusion coefficient, T2 and T1 relaxation rate of the thrombus and the plaque. A**, The percent magnetization transfer rate and **B,** the apparent diffusion coefficient were significantly different between the thrombus and the plaque and thus can be used to generate endogenous contrast between these two tissue components. **C**, The T2 and **D**, T1 relaxation rates were similar between the two components.

A summary of the % MTR, ADC, T2 and T1 relaxation times calculated for all the thrombi and plaques included in this study is presented in Figure 4. The %MTR and ADC are significantly different between the thrombus and the plaque and thus can be used to generate endogenous contrast between these two components. Conversely, the T2 and T1 relaxation times are similar between the two components, which results in a lower CNR.

### %MTR at 11.7 T correlates with the protein (fibrin and collagen) content of the thrombus

**Figure 5:**
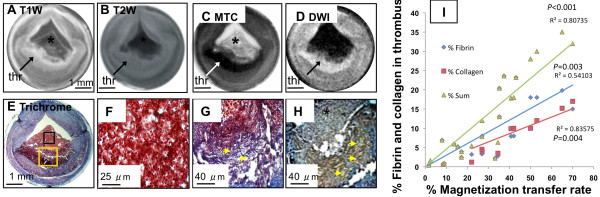
**Magnetization transfer contrast correlates with the protein (fibrin and collagen) content of the thrombus. A**, T1W image shows a hyperintense thrombus overlying the plaque. Post mortem coagulated erythrocytes are located in the center of the lumen (asterisk). **B**, T2W image in which the thrombus appears grey and coagulated erythrocytes are hypointense. **C**, MTC image identifies thrombus as hypointense and the coagulated blood as light grey. **D**, DW image identifies the thrombus as hyperintense and the coagulated blood as a signal void. **E**, Masson’s trichrome staining. **F**, Higher magnification of the coagulated blood (black box indicated in 2E) demonstrates erythrocytes interspersed within a few fibrin fibers. **G-H**, Trichrome staining (G) and fibrin immunostaining (H) taken within the thrombus (yellow box indicated in 2E) reveal a dense collagen (2G; arrowheads) and fibrin fibers (2H; arrowheads). **I**, The graph shows significant linear correlation between the % MTR and the protein content of thrombi.

Image characteristics of coagulated erythrocytes and thrombus associated with plaque disruption within the same rabbit vessel is illustrated in Figure 5. Coagulated erythrocytes, located in the center of the lumen, had a bright periphery and a darker center on the T1W image (Figure 5A) and a uniform hypointensity on the T2W image (Figure 5B). On the MTC image (Figure 5C), coagulated blood appeared light grey, indicating the presence of low protein content, and showed very low signal on the DW image (Figure 5D). Thrombus associated with plaque disruption was well differentiated from the coagulated blood, appearing hyperintense on T1W (Figure 5A) and isointense on T2W images (Figure 5B), but was not well delineated from the plaque. Conversely, thrombus was hypointense on the MTC image (Figure 5C) and hyperintense on the DW image (Figure 5D) and well discriminated from the underlying plaque. Histology verified the presence of coagulated erythrocytes interspersed in a loose fibrin network (Figure 5E and 5F) overlying a thrombus associated with plaque disruption enriched in collagen (Figure 5G) and fibrin (Figure 5H).

To investigate the relationship between the % MTR and the protein content of the thrombus, the amount of fibrin and collagen were quantified using immunohistochemistry and trichrome staining, respectively (Figure 5I). The % MTR correlated strongly with the % fibrin (*r* = 0.73, *P* = 0.003) and the % collagen (*r* = 0.9, *P* = 0.004) content of thrombi. The correlation was more significant when the % MTR was plotted versus the total protein content (fibrin and collagen) of the thrombus (*r* = 0.89, *P <* 0.001).

### Segmented MTC, collagen and fibrin images show the correlation between the detection of thrombus on MRI and histology

**Figure 6:**
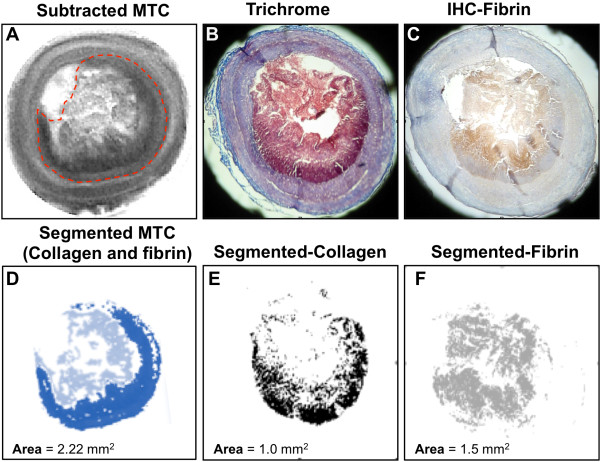
**MTC detects collagen and fibrin as seen by histology. A**, Subtracted magnetization transfer image of a vessel segment with mural thrombus (hypointense; red line). **B**, Corresponding Masson’s trichrome staining for collagen detection. **C**, Corresponding immunohistochemical staining for fibrin detection. **D-F**, Segmented MTC (D), trichrome (E), and immunohistochemistry (F) images show the correlation between MTC and histological detection of protein (collagen and fibrin) in the thrombus. MTC: magnetization transfer contrast.

Another example illustrating the correlation between the thrombus area as measured on the subtracted MT images and the corresponding histological detection of collagen and fibrin is shown in Figure 6. The subtracted MT image (Figure 6A), the Masson’s trichrome staining (Figure 6B), and the immunohistochemical staining for fibrin (Figure 6C) show the vessel wall and the luminal thrombus. The corresponding segmented images show that the hypointense region seen on the MTC image (Figure 6D) corresponds to histological regions rich in collagen (Figure 6E) and intermixed with fibrin (Figure 6F).

## Discussion

In this study, we used *ex vivo* MRI at 11.7-Tesla to evaluate the accuracy of different MRI sequences (T1W, T2W, MTC, DWI) for detecting the size and composition of thrombus associated with disrupted rabbit atherosclerotic plaques. We demonstrated that: *(i)* all sequences had sufficient SNR to visualize the thrombus, but MTC and DW images provided higher sensitivity and superior CNR to detect the thrombus compared to T1W and T2W images; *(ii)* MTC and DWI slightly underestimated the area of the thrombus (6% and 17.6%, respectively), whereas T1W and T2W images significantly overestimated thrombus area (53.7% and 46.4%, respectively) as compared to histology; *(iii)* the % MTR correlated with the protein (fibrin and collagen) composition of the thrombus. The novelty of our findings demonstrate that MT and DWI pulse sequences may be used to generate endogenous contrast to visualize thrombus and provide information about its organizational stage, without targeted contrast agents.

MTC has been used to detect intracranial hemorrhage [43,44], for imaging coronary veins [45,46], arteries [47] and myocardial perfusion [48]*in vivo* and protein-rich components of human carotid artery specimens such as the fibrous cap [37,49]*ex vivo*. However, the feasibility of MTC for thrombus imaging has not been previously explored. Here, we demonstrated that MTC could be used as an endogenous contrast mechanism to image the protein content thrombus associated with plaque disruption using rabbit aortic specimens imaged *ex vivo* at 11.7 T. We found that the subtracted MTC images increased the conspicuity between the plaque and the thrombus and allowed a more accurate detection of thrombus area. The quantitative measure of %MTR correlated with the protein content of the thrombus, permitting the distinction of platelet-rich and protein-rich rabbit thrombi (%MTR = 6.4 ± 4.2 vs. 38.6 ± 15.6). Interestingly, we found a stronger correlation between the % MTR and the collagen content of rabbit thrombi compared to their fibrin content. *In vivo* applications of MTC in human diseases other than atherosclerosis [50,51] have also reported a linear correlation between MT and tissue collagen concentration. The importance of collagen in creating an MT effect is evident by the fact that although this single macromolecule comprises only ~ 40% of the macromolecules in cartilage, it is responsible for nearly all of the MT effect observed [39,41]. Moreover, several other mechanisms contribute to the rate of MT, including the mobility, the hydration state and the number of hydroxyl and amide groups [40], as well as the concentration of the macromolecules and/or water [51].

The value of *ex vivo* DWI with low b-value (~ 450 s/mm^2^) for identifying thrombus has been demonstrated in our previous study of rabbit atherothrombosis [18] and other studies of human carotid specimens [32-35]. It has been shown that ADC, which represents the magnitude of diffusion of water molecules, varies in parallel to the phases of thrombus aging and organization [34]. An early reduction of ADC to 0.36 × 10^-3^mm^2^/s occurs after 1 week *in vitro,* consistent with the maturation of fibrin fibers, the presence of an extensive collagen network, and the entrapment of erythrocytes within this network [52]. Conversely, resolution of the thrombus results in increased water diffusion (1.33 × 10^-3^mm^2^/s). Recently it was reported that the *in vivo* ADC values of intraplaque hemmorage in carotid arteries varied from 0.48 to 1.57 × 10^-3^mm^2^/s and organized hemorrhages showed the most restricted diffusion whereas the diffusivity increased with thrombus lysing [35]. Consistently, we found that rabbit thrombi up to 3 days old undergoing the early organizational phase of thrombus evolution showed a restricted diffusion of with a ADC = 0.45 ± 0.15 × 10^-3^mm^2^/s. The importance of calculating the ADC of thrombus is that it might be used as a prognostic marker for the outcome of thrombolysis [53].

Lastly, we found that MTC and DWI at 11.7 T detected the presence of thrombus in rabbit samples with higher sensitivity and resulted in a more accurate calculation of the thrombus area than the T1W and T2W images. The lower CNR between the thrombus and the vessel wall observed on T1W and T2W images resulted in a significant over-estimation of the thrombus area and lower intra-rater reproducibility. Similar findings were reported in other studies [54] in which thrombus appeared larger on T1W images due to the presence of hemosiderin, which enhances the relaxation of protons in nearby tissues causing these regions to appear dark like the thrombus itself. It was also reported that T2W images showed the lowest sensitivity for thrombus detection [55].

### Limitations

Because the rabbit aortas were fixed in formalin, collagen residues were modified with new cross-links**.** Although little is known about the effects of formalin-fixation on the MR properties of tissues, in cartilage it results in a significant decrease of T1, T2 relaxation times, and ADC, and an increase of the % MTR [56]. However, other studies of multiple sclerosis specimens have shown a reduction of MTR [57] while the ADC of gray matter remained more or less constant [58] after formalin fixation. Although the experiments with the rabbit plaques were performed at high field, where the MT effect is greater because of the longer relaxation time of water, and in the absence of physiological motion, previous studies have shown the feasibility to apply these pulse sequences *in vivo* at clinically relevant fields. MT has been used for imaging coronary veins [45,46], arteries [47] and myocardial perfusion [48] whereas DWI has been applied for imaging human carotid atherosclerosis [34,35].

## Conclusions

We demonstrated that *ex vivo* MTC and DWI MRI at 11.7 T increased the conspicuity of thrombus and more accurately estimated the thrombus area compared to T1W and T2W images the rabbit aortic specimens. The quantitative measure of % MTR correlated with the protein content of the thrombus and allowed the discrimination between protein-rich (organized) and unorganized (cell-rich) thrombus since organized thrombus elicited the higher %MTR. Our *ex vivo* data demonstrate a new potentially valuable utilization of MT and DWI MRI for non-invasive imaging of thrombus without targeted contrast agents. Application of these techniques for *in vivo* thrombus imaging may allow detection of vascular occlusion due to atherosclerotic plaque rupture, and monitoring the effectiveness of antithrombotic, anti-platelet and thrombolytic agents.

## Competing interests

The authors declare that they have no competing interests.

## Authors’ contributions

AP carried out all the procedures related to the animal model performed all the CMR scans, analyzed the acquired CMR and histological data, contributed to the statistical analyses, obtained all illustrations, wrote the manuscript, and merged all feedback from the co-authors into the final manuscript. QY: Optimized the data acquisition parameters for the MTC at 11.7 T. NG: Was the independent observer for the analysis of the thrombus area on the MR images and histological data. JAH: Revised the manuscript critically. All authors have read and approved the final manuscript.
